# Prevalence of Obesity and Metabolic Syndrome in Children with Type 1 Diabetes: A Comparative Assessment Based on Criteria Established by the International Diabetes Federation, World Health Organisation and National Cholesterol Education Program

**DOI:** 10.4274/jcrpe.galenos.2019.2019.0048

**Published:** 2020-03-19

**Authors:** Özlem Yayıcı Köken, Cengiz Kara, Gülay Can Yılmaz, Hasan Murat Aydın

**Affiliations:** 1 University of Health Sciences Turkey, Dr. Sami Ulus Training and Research Hospital, Clinic of Pediatric Neurology, Ankara, Turkey; 2 İstinye University Faculty of Medicine, Department of Pediatrics, İstanbul, Turkey; 3 Mardin State Hospital, Clinic of Pediatric Endocrinology, Mardin, Turkey; 4 Ondokuz Mayıs University Faculty of Medicine, Department of Pediatric Endocrinology, Samsun, Turkey

**Keywords:** Type 1 diabetes, metabolic syndrome, double diabetes, prevalence

## Abstract

**Objective:**

To determine the prevalence of obesity and metabolic syndrome (MetS) in children and adolescents with type 1 diabetes (T1D) and to compare the widely accepted and used diagnostic criteria for MetS established by the International Diabetes Federation (IDF), World Health Organisation (WHO) and National Cholesterol Education Program-Adult Treatment Panel III (NCEP-ATPIII).

**Methods:**

We conducted a descriptive, cross sectional study including T1D patients between 8-18 years of age. The three sets of criteria were used to determine the prevalence of MetS and findings compared. Risk factors related to MetS were extracted from hospital records.

**Results:**

The study included 200 patients with T1D (52% boys). Of these, 18% (n=36) were overweight/obese (body mass index percentile ≥85%). MetS prevalence was 10.5%, 8.5% and 13.5% according to IDF, WHO and NCEP criteria, respectively. There were no statistically significant differences in age, gender, family history of T1D and T2D, pubertal stage, duration of diabetes, hemoglobin A1c levels and daily insulin doses between patients with or without MetS. In the overweight or obese T1D patients, the prevalence of MetS was 44.4%, 38.8% and 44.4% according to IDF, WHO and NCEP-ATPIII criteria, respectively.

**Conclusion:**

Obesity prevalence in the T1D cohort was similar to that of the healthy population of the same age. Prevalence of MetS was higher in children and adolescents with T1D compared to the obese population in Turkey. The WHO criteria include microvascular complications which are rare in childhood and the NCEP criteria do not include a primary criterion while diagnosing non-obese patients according to waist circumference as MetS because the existence of diabetes is considered as a direct criterion. Our study suggests that IDF criteria which allows the diagnosis of MetS with obesity and have accepted criteria for the childhood are more suitable for the diagnosis of MetS in children and adolescents with T1D.

**What is already known on this topic?**

Some studies have reported the prevalence of obesity and metabolic syndrome (MetS) in pediatric patients with type 1 diabetes (T1D). There are no such data from Turkey. In T1D patients diagnosis of MetS at early ages is critical to manage and prevent macrovascular complications. Nevertheless, identifying the presence of MetS in T1D is difficult and it is not clear which criteria are most suitable for accurate identification.

**What this study adds?**

The prevalence of obesity and MetS in children with T1D in our region is reported. MetS prevalence was 10.5%, 8.5% and 13.5% according to International Diabetes Federation (IDF), World Health Organisation (WHO) and National Cholesterol Education Program (NCEP) criteria, respectively. Also, this study comparatively assesses the widely accepted and used diagnostic criteria for MetS established by IDF, WHO and NCEP. Using IDF criteria seems more suitable because obesity is a prerequisite and they include accepted criteria for childhood.

## Introduction

Type 1 diabetes (T1D) is a chronic disease characterized by absolute insulin deficiency due to immune-mediated destruction of pancreas beta cells. T1D has been associated with a leaner phenotype in the past. However, the number of obese patients with T1D is increasing, mirroring the global increase in obesity prevalence (1,2). Metabolic syndrome (MetS), also called insulin resistance syndrome, comprises a cluster of diagnostic criteria including abdominal obesity, type 2 diabetes (T2D) and cardiovascular risk factors such as hypertension, dyslipidemia and nephropathy (3). Determining the frequency of MetS in patients with T1D requires the determination of coexisting obesity, hypertension and, dyslipidemia in these patients. Yet, studies in pediatric T1D patients to determine the prevalence of obesity and MetS are scarce.

In the T1D population, while the incidence of microvascular complications is decreasing owing to intensive diabetes management, macrovascular complications are now more commonly seen, a finding considered to be associated with the increasing incidence of obesity and MetS (4,5,6,7,8). This has led to the realisation that diagnosis of MetS and obesity is essential if continued improvements in the quality and duration of life in children and adolescents with T1D is to be achieved. For the diagnosis of MetS in childhood and adolescence, the International Diabetes Federation (IDF) criteria are globally accepted. The World Health Organization (WHO) and the National Cholesterol Education Program-Adult Treatment Panel III (NCEP-ATPIII) criteria used for the diagnosis of MetS in the adult population can also be used for children and adolescents, with some modifications. The aim of this study was to determine the prevalence of obesity and MetS in a large cohort of children and adolescents with T1D, and to compare widely accepted and used criteria for the diagnosis of MetS.

Ethics committee approval was received for this study from the Local Ethics Committee of the Faculty of Medicine, Samsun Ondokuz Mayıs University (2014-354).

## Patients and Data Documentation

A descriptive, cross sectional study was conducted, comprising a total of 200 T1D patients between 8-18 years of age who were followed up for at least six months. Clinical and laboratory data were obtained from the patients’ medical records, including age, sex, anthropometric measurements, duration of diabetes, daily insulin dose (IU), degree of metabolic control based on mean annual hemoglobin A1c (HbA1c) values, comorbidities and treatments. History of T1D and/or T2D in first- and second-degree relatives was recorded. Laboratory results were collected which included HbA1c, total cholesterol, high density lipoprotein (HDL), low density lipoprotein (LDL) and triglyceride concentrations. Average IU per kg of body weight was calculated for the intensive insulin treatment group. Briefly, the total IU over three randomly selected days from the previous month was collected and mean daily dose was divided by body weight. The patients were evaluated for complications of diabetes and accompanying diseases. Existence of hypertension, prehypertension, microalbuminuria, retinopathy and neuropathy in addition to thyroid and coeliac disease were recorded. Patients who had other types of diabetes including T2D, maturity onset diabetes of the young and secondary diabetes were excluded from the study.

Height, weight and waist circumference (WC) were measured and body mass index (BMI) was calculated. Overweight and obesity were defined as a BMI ≥85^th^ and ≥95^th^ percentile, respectively (9).

The existence of hypertension was defined as a positive history of antihypertensive medicine or average blood pressure measurements above the 95^th^ percentile of Turkish pediatric age measurements determined by Tümer et al (10). Values between 90 and 95^th^ percentile were accepted as prehypertension while values below the 90^th^ percentile were accepted as normal. The amount of albumin measured in appropriately collected 24-hour urine samples was used to determine the existence of nephropathy. Albumin less than 30 mg in 24 hours was considered negative and more than 30 mg was considered positive for albuminuria. The existence of pathologic changes indicating retinopathy in ophthalmoscopy were recorded. The patients were questioned for coeliac disease and the scans were completed using laboratory tests when necessary.

Total daily insulin dosage for the last three months was obtained by randomly selecting three days from the patient records. Mean daily dose (IU/kg) was calculated by dividing total daily IU to patient weight (kg).

In order to establish the degree of metabolic control, mean HbA1c level, measured over the past year, was calculated. The patients were divided into three metabolic control groups based on mean annual HbA1c: good, HbA1c <7.5%; moderate, HbA1c 7.5-9%, and poor control, HbA1c >9% (1).

The patient groups whose anthropologic and clinical data were recorded at first presentation, the third month of follow-up and the last routine clinic visit consisted mainly of patients who had ketoacidosis at presentation and patients who had just begun treatment. For this reason, data obtained after the onset of disease and at the third month of treatment were accepted as baseline values. The evaluation at the last routine clinic visit was deemed to represent outcomes under treatment.

WHO and NCEP-ATPIII diagnostic criteria were applied to the study cohort to determine the prevalence of MetS in addition to the IDF MetS criteria (2005) for children and adolescents. WHO defines MetS as glucose intolerance, impaired glucose tolerance or diabetes mellitus, and/or insulin resistance, along with two or more of the following: high blood pressure (≥140/90 mmHg); hypertriglyceridemia (≥150 mg/dL); and/or low HDL cholesterol (<35 mg/dL in men and <39 mg/dL in women); central obesity (waist/hip ratio >0.9 in men and >0.85 in women); and/or a BMI >30 kg/m^2^ and microalbuminuria (urinary albumin excretion rate ≥20 µg/min or albumin/creatine ratio ≥30 µg/mg) (11). According to the NCEP-ATPIII definition, a subject has MetS if they meet three or more of the following criteria: abdominal obesity (WC ≥102 cm in men and ≥88 cm in women); hypertriglyceridemia (≥150 mg/dL); low HDL cholesterol (<40 mg/dL in men and <50 mg/dL in women); high blood pressure (>130/85 mmHg); and/or high fasting glucose (>110 mg/dL) (12). Children over eight years of age were eligible for the study because there were well-defined criteria to diagnose MetS in children aged six to 10 years and above 10 years (13). According to the IDF definition of MetS in children a subject has MetS if he or she is between 6 to 10 years of age and has obesity defined as having a WC >90^th^ percentile. If the age is between 10 to 16 years, a subject has MetS if he or she has a WC value >90^th^ percentile (or adult cut-off, if lower) and has two or more of the following criteria: hypertriglyceridemia (≥150 mg/dL); low HDL cholesterol (<40 mg/dL); high blood pressure percentile for age, sex and height; and/or raised fasting glucose (>100 mg/dL). Since WHO and NCEP-ATPIII criteria were formulated for adults, for the purposes of this study these criteria were modified for use in our study cohort by applying pediatric percentiles All children with T1D were assumed to have impairment of glucose tolerance and fasting high blood sugar. Impaired glucose tolerance, impaired fasting glycemia or existence of T2D, which are part of the mentioned criteria were accepted as positive for our T1D patient group. Patients were examined for the existence of either of the two remaining criteria. Dyslipidemia was accepted as a HDL concentration <50 mg/dL and triglyceride >150 mg/dL. Pediatric percentiles were used for estimation of hypertension, WC and BMI (9). Patients with MetS according to the IDF criteria were compared in terms of demographic and clinical data.

### Statistical Analysis

Data analyses were performed by using SPSS for Windows, version 22.0 (SPSS Inc., Chicago, IL, United States). Whether the distribution of continuous variables were normal or not was determined by Kolmogorov-Smirnov test. Levene test was used for the evaluation of homogeneity of variances. Unless specified otherwise, continuous data were described as mean±standard deviation (SD) for normal distributions, and median (range) for skewed distributions. Categorical data were described as number of cases (%).

Statistical analysis differences in normally distributed variables between two independent groups were compared by Student’s t-test, Mann-Whitney U test were applied for comparisons of the not normally distributed data. While the differences in normally distributed variables among more than two independent groups were analyzed by one-way ANOVA, otherwise, Kruskal-Wallis test was applied for comparisons of the not normally data. When the p value from one-way ANOVA or Kruskal-Wallis test statistics were statistically significant post-hoc LSD or Conover’s non-parametric multiple comparison test were used to know which group differ from which others.

## Results

The study group consisted of 200 T1D patients with a mean age of 13.8±2.8 years, duration of diabetes 4.6±3.3 years. More than half (52%) of the patients were male and the majority (87%) pubertal. Mean HbA1c was 8.40±1.63% and mean IU was 0.87±0.26 U/day. In the family history, T1D and T2D were present in 17.5% and 44% of the patients, respectively. Only three patients were using an insulin pump, and all the remaining patients (n=197) were on multiple insulin injections. Metabolic control (mean annual HbA1c) across the whole cohort was good in 26.5%, moderate in 37% and poor in 36.5%. Of the 200 patients with T1D, 19 (9.5%) were overweight and 17 (8.5%) were obese.

**Figure 1:**
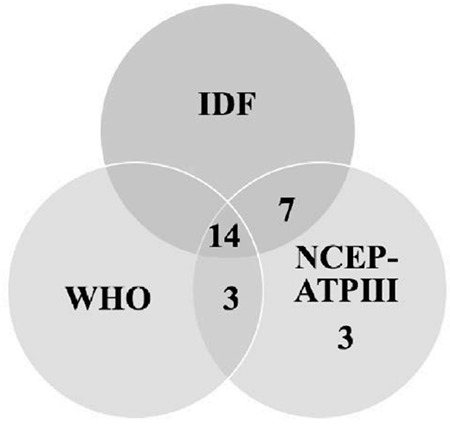
The numbers of patients with metabolic syndrome diagnosis based on different diagnostic criteria IDF: International Diabetes Federation, WHO: World Health Organisation, NCEP-ATPIII: National Cholesterol Education Program-Adult Treatment Panel III

Prevalence of MetS in the whole study cohort was 10.5%, 8.5% and 13.5% according to IDF, WHO and NCEP-ATPIII criteria, respectively. Figure 1 shows a Venn diagram of the numbers of patients with MetS diagnosis according to different diagnostic criteria for whole group. However, in the 36 overweight/obese T1D patients, the prevalence of MetS was 44.4%, 38.8% and 44.4% according to IDF, WHO and NCEP-ATPIII criteria, respectively.

Table 1 depicts the clinical characteristics of MetS positive versus negative T1D patients according to IDF criteria. There were no statistically significant differences in age, gender, family history of T1D, pubertal stage, duration of diabetes, HbA1c levels and daily IU between patients with or without MetS but the difference was significant concerning family history of T2D and clinical and laboratory components of MetS. LDL-cholesterol and triglyceride concentrations were significantly elevated in patients with MetS (p<0.001). When demographic and clinical data of patients with and without MetS according to WHO and NCEP-ATPIII criteria were evaluated, similar results to IDF criteria were obtained (data not shown).

As shown in Table 2, BMI-SD score (SDS) values of all patients increased during intense insulin treatment following diagnosis according to the IDF, WHO and NCEP-ATPIII criteria. The BMI-SDS values of the group diagnosed with MetS according to IDF, WHO and NCEP-ATPIII criteria, were significantly greater than the non-MetS group in all three evaluations. All cases diagnosed as MetS according to all three sets of criteria had BMI values above the 50^th^ percentile at the diagnosis of T1D.

## Discussion

There is no data on the prevalence of obesity in children and adolescents with T1D in Turkey. In our study group, comprising 200 children with T1D between 8 and 18 years, total prevalence of overweight and obesity was found to be 18%. The prevalence of overweight and obesity in schoolchildren and adolescents from different regions of our country has been reported in the range of 12.8-20.2% (14,15,16,17,18). Accordingly, the present study has shown that obesity prevalence of our T1D cohort was similar to that of the general population in Turkey. Studies from other countries have indicated that the increased incidence of overweight and obesity in T1D population mirrors what happens in the general population (19,20,21,22,23). Although the traditional belief is that patients with T1D are normal or thin, overweight and obesity figures in these patients have been found to increase in parallel with the normal population. Intense insulin therapy and weight gain due to the anabolizing and lipogenic effect of insulin are thought to be responsible for the increase. Additionally, the change in nutrition habits and shift to sedentary life style which are probably responsible for a global increase in overweight and obesity have also affected the young population with T1D. At present, T1D patients are more obese compared to the past, which has been associated with intense insulin treatment. The Epidemiology of Diabetes Interventions and Complications study revealed that the incidence of obesity in T1D patients has significantly increased due to widespread intense insulin treatment following Diabetes Control and Complications Trial (23,24,25). Some authors indicated that being female was a risk factor for a higher BMI-SDS six years after diabetes onset (26). All of our patients had gained weight at the time of diagnosis, third month of follow up and the last visit. Since the patients probably lost weight before diagnosis, the BMI-SDS values at the third month follow up and the last visit were evaluated and a striking weight gain was present. This reflects the effect of intense insulin treatment. All the patients gained weight but weight gain was more pronounced in the MetS positive group. Another striking point is that the BMI and BMI-SDS values in the MetS positive group were significantly higher than the MetS negative group and this was present from diagnosis up until the study period.

Data on MetS prevalence in patients with T1D is controversial. The prevalence of MetS was evaluated using three sets of recognized criteria and in 200 T1D children; it was 10.5% according to IDF, 8.5% according to WHO and 13.5% according to NCEP-ATPIII. Although there are no studies that report the prevalence of MetS in children and adolescents with T1D in Turkey, MetS seems to be more common in the T1D population when compared to the obese population. The results of the small number of studies comparing the prevalence of MetS in T1D children and adolescents vary between countries. In a study where 115 T1D patients between 5-16 years of age were investigated, MetS prevalence was found to be 13.2% according to IDF. The researchers found a significantly low incidence of MetS for that study population (27,28). Pinhas-Hamiel et al (20) reported a 7.1% prevalence of MetS in 326 T1D patients whose median age was 18.5 years.

The prevalence of MetS in overweight and obese children and adolescents in Turkey has been reported to vary between 20%-38% according to WHO, IDF and NCEP-ATPIII criteria (29,30,31). In our study, the incidence of MetS in overweight and obese patients was 41.7% according to IDF, 38.3% according to WHO and 47.2% according to NCEP-ATPIII criteria which is somewhat higher than has previously been reported but may simply reflect the secular trend in increasing prevalence of overweight and obesity.

Although it is widely accepted that it is necessary to diagnose MetS in the early stages of T1D, the main problem is the inadequacy of the criteria for the diagnosis of MetS in T1D patients. Use of the NCEP-ATPIII criteria resulted in the highest prevalence in our study (13.5%) since NCEP-ATPIII allows diagnosis of MetS by any three positive out of five criteria, with no mandatory prerequisites. Since diabetes is accepted as positive, two out of the remaining four criteria are enough and these criteria are already present in patients diagnosed using IDF criteria. As such, the MetS positive group determined by NCEP-ATPIII includes both the IDF criteria group and the group which did not meet the WC criterion, that is they are not obese according to WC, but are positive for two other criteria. The WC of the group which meets NCEP-ATPIII criteria but not the IDF criteria is below 90^th^ percentile while the two criteria they met were any two of hypertriglyceridemia, low HDL or hypertension. Since there are no primary criteria in NCEP-ATPIII and it evaluates the WHO dyslipidemia criteria as two distinct criteria, it encompasses all cases diagnosed using WHO and IDF.

When the results of our study were evaluated according to IDF criteria, it was evident that the incidence of MetS was higher in the group where T1D was accompanied by overweight and obesity when compared to those who do not have T1D but are overweight or obese. The incidence of MetS is similar in overweight and obese individuals independent of T1D, according to WHO criteria. This discrepancy is caused by differences in definitions of MetS. Impaired glucose tolerance or T2D is a prerequisite for MetS in the WHO criteria, so it is understood that MetS prevalence will not change if the criteria is actualized as T1D. On the other hand, obesity as determined by WC is a prerequisite for MetS while impaired glucose tolerance and T2D are secondary criteria. Since this criterion is met by all patients in addition to obesity in our study population, which consisted of T1D patients, the ratio was found to be high.

On the other hand, insulin resistance and/or diabetes are mandatory primary criteria for the WHO definition and our T1D patient group meet these criteria. The WHO criteria also include microalbuminuria, which has the highest sensitivity for MetS in adulthood. However, our study group, because of the patient age range, had the lowest prevalence for this criterion, since these complications had not yet occurred (32,33,34). In the study group, there were 25 patients whose BMI was below the 95^th^ percentile while WC was above the 90^th^ percentile, which led to the identification of more MetS using IDF and NCEP-ATPIII in T1D. All patients who were diagnosed positive using the WHO criteria are found to also meet NCEP-ATPIII.

The WHO criteria include microvascular complications, which are rare in childhood, and the NCEP criteria do not include a primary criterion while diagnosing non-obese patients according to WC as MetS because the existence of diabetes is considered as a direct criterion. Due to these reasons, these criteria do not seem to be useful for the diagnosis of MetS in children and adolescents with T1D. Using IDF criteria seems more suitable because obesity is a prerequisite and they include accepted criteria for childhood (11,12,13).

### Study Limitations

Our study has several limitations and strengths. The main limitation is the absence of accepted clinical and laboratory criteria for the diagnosis of MetS in children and adolescents with T1D. Thus existing criteria had to be modified for a pediatric population in order to determine the prevalence. On the other hand, using and comparing three different modified criteria is a strength of our study. A further strength of this study lies in the accuracy of data, which adds to the available information concerning the prevalence of MetS in children and adolescents with T1D.

## Conclusion

Overweight and obesity prevalence of the group with T1D was similar to that of the population of the same age group in Turkey, but the prevalence of MetS was found to be higher than that of the general population. Except for the components of MetS, the other clinical and laboratory parameters were not helpful for prediction. It has been observed that all children and adolescents with T1D gained weight under intense insulin treatment. However, weight gain was more prominent in the MetS positive group. It is clear that appropriate modification of the criteria is required for the early detection of MetS in children and adolescents with T1D. This study suggests that IDF criteria are more suitable for the diagnosis of MetS in children and adolescents with T1D.

## Figures and Tables

**Table 1:**
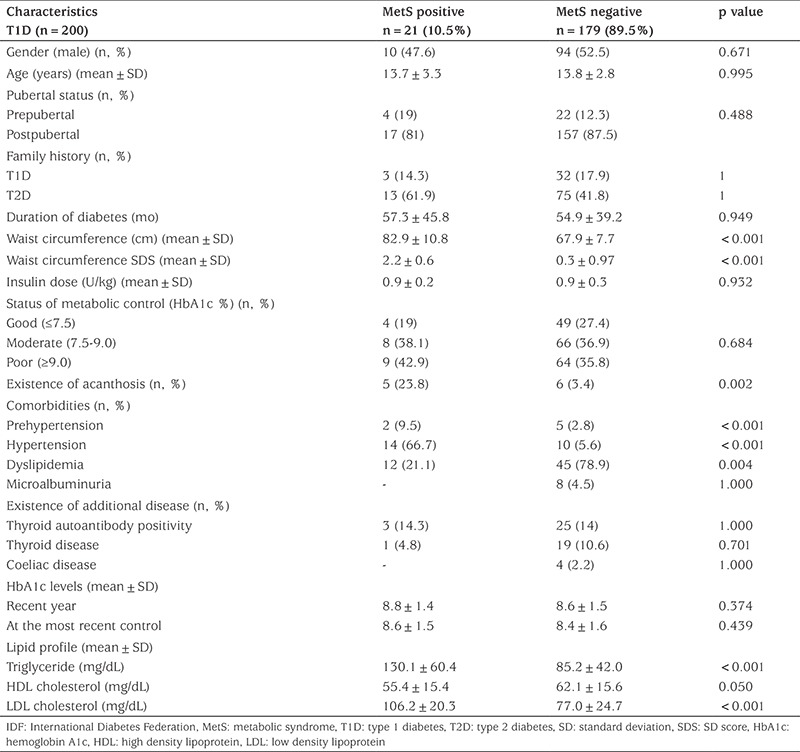
Comparison of the demographic and clinical findings of type 1 diabetes patients with and without metabolic syndrome according to International Diabetes Federation criteria (10)

**Table 2:**
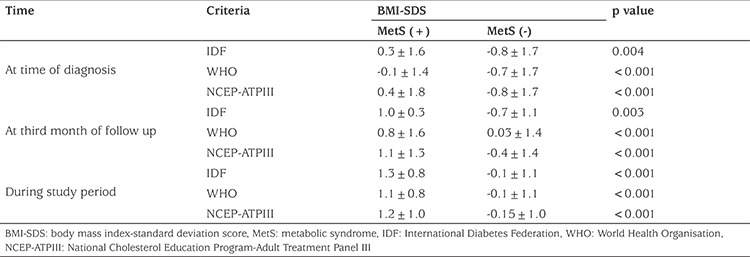
Changes in body mass index-standard deviation score values over time among metabolic syndrome positive and negative groups according to International Diabetes Federation, World Health Organisation and National Cholesterol Education Program-Adult Treatment Panel III criteria
